# Effectiveness of Povidone-Iodine 1% Eye Drop on *Streptococcus pneumoniae* and *Escherichia coli* Induced-Keratitis in Mice

**DOI:** 10.22086/gmj.v8i0.1161

**Published:** 2019-06-02

**Authors:** Mahsa Hadipour Jahromy, Abdolhamid Najafi, Farzaneh Majdi Nassab, Mahya Moiniyan

**Affiliations:** ^1^Herbal Pharmacology Research Center, Department of Pharmacology, Faculty of Medicine, Islamic Azad University, Tehran Medical Sciences, Tehran, Iran; ^2^Department of Ophthalmology, Faculty of Medicine, Islamic Azad University, Tehran Medical Sciences, Tehran, Iran

**Keywords:** Streptococcus pneumoniae, Keratitis, Cornea, Escherichia coli, Mouse

## Abstract

**Background:**

Bacterial keratitis is an ophthalmic infection that may result in irreversible corneal damage. This study aimed to examine the effectiveness of povidone-iodine eye drop 1% in eye infection caused by inoculation of *Streptococcus pneumoniae* and *Escherichia coli* of mice.

**Materials and Methods:**

In this study, 49 adult male CBA/J mice were used that divided into seven equal groups. The corneas of all mice were scratched and infected with a clinical strain of either *S. pneumoniae* or *E. coli* topically, except control group. Subgroups received chloramphenicol 0.5% eye drop twice daily in case of *S. pneumoniae* infection or ciprofloxacin 0.3% eye drop every 4 hours following *E. coli* infection from or povidone-iodine 1% eye drop in both groups, from post infection (PI) day 3 to7. Slit lamp examinations (SLE) of the corneas and eyes were performed every day to examine detectable or intense corneal opacity and erosion.

**Results:**

In all infected mice, SLE scores were significantly higher than the control group on PI day 3. Scores increased steadily by time in all infected groups without treatment, reached to maximal value on PI day 7. In infected groups, treatment with either povidone-iodine 1% or chloramphenicol 0.5% or ciprofloxacin 0.3% on day 3, significantly decreased the SLE scores on PI day 7.

**Conclusion:**

Povidone-Iodine 1% was effective to decrease *S. pneumoniae* and *E. coli* induced-keratitis symptoms in mice. Treatment with povidone-iodine 1% was observed time-dependently and was comparable to common eye drop antibiotics.

## Introduction

Conjunctivitis is a common ophthalmic infection that most frequently seen by general practitioners. In addition to the annual cost for medical care, even with treatment, in some cases these infections can cause blindness and disability. *Streptococcus pneumoniae* is among opportunistic bacteria that causes keratitis and may result in irreversible corneal damage [1-5]. Topical antimicrobial treatment almost always is effective for superficial pneumococcal eye infections [2-3]. Although* Escherichia coli* is rarely found in the normal flora of the conjunctiva, it is the cause of around 5-10% of endogenous bacterial endophthalmitis [2]. It is usually seen as a source of infection in ophthalmic neonates.Antimicrobial resistance may occur, and its prognosis is very poor; hence, early diagnosis and treatment are dramatically necessary to keep appropriate vision [2]. These infections very commonly occur in those who are immunocompromised, diabetic and/or in corneas with an existing pathologic condition. Exogenous endophthalmitis is associated usually with intraocular surgery or trauma [5]. In endogenous endophthalmitis, the most common primary site of infection was urinary tract, and most patients have diabetes. Early recognition and suitable treatment are vital because of its poor prognosis. Depending on the severity, most patients need immediate management and intervention [6]. Topical application of antiseptic agents to prevent blindness evolved some experiences. Today povidone-iodine can be used throughout the world to prevent many harmful infections [6]. Following the use of iodine solution to the body, a reduction in eye flora was reported and therefore the possible effect of iodine on the eye was announced in 1951. Then, iodophors were reported to do so in 1970, and later on, the specific combination of povidone and iodine was applied for direct ophthalmic indication [7]. Some of its valuable advantages are being nontoxic, inexpensive, broad spectrum, and finally easily available [5-7]. However, few reports have been published about povidone-iodine effectiveness in the treatment of microbial pathogens [8, 9]. The existing reports are neither accurate according to specific pathogen nor valid considering patient sample size. They have just mentioned one of the povidone-iodine off-label indications. Therefore, in this study, we investigated in detail; the therapeutic effects of a solution of povidone-iodine 1% in eye infection caused by inoculation of either *S.pneumoniae or E. coli* in mouse eyes and compared them with effects of common antibiotics.

## Materials and Methods

### Animals

Forty-nine adult CBA/J mice weighing 20-23 g were purchased from Pasteur Institute (Iran) and housed under laboratory conditions: temperature 23±1°C, humidity 40-60%, 12h: 12h light/dark cycle, lights on at 07:00h. Food and water were available *ad libitum*. All the experiments were carried out between 10:00 and 15:00 in testing rooms adjacent to the animal rooms. Mice were treated in accordance with the current law and the NIH Guide for the Care and Use of Laboratory. The ethical and legal approval for animal experiments of the present study has been obtained from Research and technology Deputy of Azad University, Tehran Medical Sciences Branch, Tehran, Iran (Register NO.40730.1394).

### Drugs

Povidone-iodine 1% were obtained from Behsa Company (Iran). Chloramphenicol 0.5% and ciprofloxacin 0.3% eye drops purchased from Sina-Darrou Pharmaceutical Company (Iran).

### Groups and Design

In this study, 49 adult male CBA/J mice were divided into seven equal groups. The corneas of all mice were scratched and infected with a clinical strain of either *S. pneumoniae* or *E. coli* topically, except control group. Group A divided into three subgroups: one remained without any treatment while infected with *S. pneumoniae* and subgroups 2 and 3 treated with either povidone-iodine 1% or chloramphenicol 0.5% eye drop twice daily on post-infection (PI) day 3 to7. Group B divided into 3 subgroups: one remained without any treatment while infected with *E. coli* and subgroups 2 and 3 treated with either povidone-iodine 1% or ciprofloxacin 0.3% eye drops every 4 hours daily from on PI days 3 to7.

### Procedures

*S. pneumonia*e and *E. coli* were provided by the Microbiology Laboratory at Faculty of Medicine at Azad University. To induce mice eyes infection, all animals were anesthetized with appropriate doses of xylazine (Alfasan, Netherland) and ketamine (Rotex Medica, Germany). A 28- gauge needle was used to scratch the corneal surface in 3 parallel lines without penetrating the superficial stroma. To each scratched cornea, 5 microliters aliquot containing 1 × 105 CFU (Colony Forming Unit) was applied [10]. Scratch-only controls did not receive bacteria.

### Slit lamp examinations (SLE)

The SLE of the corneas and eyes using bio-microscope (Topcon, Japan) were performed to examine redness, inflammation, and secretion on PI days 1, 3, 5 and 7. The parameters used were as follows: 0 = clear/ normal; +1 = mild opacity that can be detected; +2 = moderate or dense opacity; +3 = severe opacity covering the entire corneal layer/ corneal erosion [10]. Scores were expressed as the means ± standard errors of the mean.

### Statistical Analysis

Data were analyzed using Origin 6 analytical software (OriginLab Corporation). Data were expressed as means ± the standard errors of the mean. Morphological SLE scores were analyzed using nonparametric one-way analysis of variance (ANOVA). P<0.05 was considered significant.

## Results

All mice in the control groups A and B were examined by SLE at defined time points. In the only-scratched mice in the control groups, no corneal opacity was observed. All infected mice showed no changes in their infected eye and the corneas were clear/ normal on the first day of PI. However, by PI day 3, mild corneal opacity was started and observed in all infected mice. SLE scores increased daily in the untreated groups and reached to maximum on PI day seven (Table-1 and 2).

### SLE Score of Infected Mice with S. pneumoniae

Mean score in the infected group without treatment increased by the time reached to 2.75 ± 0.15 by PI day 7 compared to the control group (P<0.001, Table-1). Treatment with either povidone-iodine1% or chloramphenicol started on PI day 3. Significant reduction in SLE scores reported on PI days 5 and 7, time-dependently (0.35± 0.08 for the povidone-iodine-treated group and zero for the chloramphenicol-treated group, on PI day7), when compared to the infected, untreated group (P<0.001 and P<0.01, respectively).

### SLE Score of Infected Mice with E. coli

Mean score in the infected group without treatment increased by the time reached to 2.50 ± 0.20 by PI day 7 when compared to the control group (P<0.001, Table-2). Application of either povidone-iodine 1% or ciprofloxacin 0.3% eye drop started on PI day 3. Significant reduction in SLE scores reported on PI days 5 and 7, time-dependently, compare to the infected group (0.30± 0.12 for povidone-iodine treated group and zero for the ciprofloxacin-treated group on PI day 7).

## Discussion

In this study, we demonstrated that povidone-iodine 1% eye drop is effective in treating an ongoing ocular infection induced by *S. pneumoniae* and *E. coli* in mouse strain that were fully susceptible to pneumococcal keratitis. In a previous study, it has been reported that the CBA/J mouse showed signs and symptoms of corneal infection beginning on PI day 3 and continuing to PI day 10 [7, 8]. The disease has been confirmed based on both histopathology and SLE scores that BALB/c and C57BL/6 mice were not susceptible to this infection under the experimental conditions that were performed. In fact, this is one of the limitations of corneal infection studies that only particular strain of mouse can be used. The infected corneas showed considerable existence of PMNs in the stroma, opacity, and disruption of the epithelium [9]. It was reported that bacterial colonization, host cellular responses, and bacterial virulence factors account for the pathogenesis of pneumococcal keratitis, which results in damaging inflammatory response [10].Although some papers have been published about the use of povidone-iodine to prevent ocular infections [5-9], no research has been reported the effectiveness of povidone-iodine 1% on *S. pneumonia*e and *E. coli* and compare to specific topical antibiotic therapy. The results of the present study showed that the rate of decrease in SLE scores was higher when specific antibiotics used topically. However, it was not statistically significant when compared to the povidone-iodine-treated groups. Besides, the onsets of action for all treatments were the same. Povidone-iodine has been known to have many potential therapeutic effects for decades. It has also been considered as part of the preoperative preparation of the eye in cataract surgery [4]. It has been reported that povidone-iodine could significantly reduce the bacterial ocular flora in normal neonates [6]. According to Isenberg *et al*. study, when antibiotic treatment is not practical, povidone-iodine 1.25% can be considered for the treatment of bacterial keratitis [7]. However, this study has some major problems. On the other hands, their trial was performed only in two countries (India and Philippine) with small sample size. Besides, based on that publication, there is not a good reason to consider povidone-iodine ocular solution as a drug of choice for bacterial keratitis, and there is no novelty on it. Based on the present study, the effectiveness of povidone-iodine 1% is confirmed, and it is proposed that povidone-iodine can be considered as first-line therapy in developing areas that *S. pneumonia*e or *E. coli* infection can result in corneal scarring and blindness. Povidone-iodine 1% was chosen in this study because it is not toxic to eye in appropriate concentrations. Povidone-iodine has a very broad antimicrobial spectrum including bacteria, viruses, and fungi and has enough contact time in vitro [9]. Bacteria resistance has rarely happened so far. Although povidone-iodine turns the eye brown for a few minutes, it is easily available as a solution or powder, and so it is widely available throughout the world in some form. Finally, the most valuable point for use in developing areas is that it is not expensive [5-10].

## Conclusion

Finally, povidone-iodine 1% is effective to attenuate *S. pneumonia*e and *E. coli* induced- keratitis symptoms in A/J mice. Treatment has been achieved within 5 days that was comparable to common eye drop antibiotics. Therefore, it can be introduced as an appropriate first-line agent for bacterial keratitis treatment.

## Acknowledgment

The research was supported by the grant (No. 40730) from the Research Deputy of Islamic Azad University, Tehran Medical Sciences, Tehran, Iran.

## Conflict of Interest

There is no conflict of interest among authors.

**Table 1:** SLE Scores from PI Days 1 to 7 Among Group A

**PI Days**	**Control group** ^1^	**Infected group** ^2^	**Infected + povidone-iodine 1% group**	**Infected + chloramphenicol group**
**Day 1**	0.09 ± 0.01	0.12 ±0.02	0.15 ±0.02	0.20 ±0.04
**Day 3**	0.22 ±0.02	1.25 ±0.07 ^b^	1.43 ±0.13^b^	1.33 ±0.07^b^
**Day 5**	0.35 ±0.05	1.95 ±0.09^c^	0.75 ±0.05^b,d^	0.55 ±0.05^a,d^
**Day 7**	0.31 ±0.04	2.75 ±0.15^c^	0.35 ±0.09^d^	0 ±0.00^e^

^1^ Not-infected only scratched ^2^ Infected by S. pneumoniae without treatment ^a^ <0.05, ^b^P<0.01, ^c^P<0.001 vs. control group; ^d^ P<0.01, ^e^ P<0.001 compared to only infected groups.

**Table 2:** SLE Scores from PI Days 1 to 7 Among Group B

**PI Days**	**Control group** ^1^	**Infected group** ^2^	**Infected + povidone-iodine 1% group**	**Infected + ciprofloxacin 0.3% group**
**Day 1**	0.05 ± 0.01	0.03 ±0.01	0.05 ±0.02	0.03 ±0.01
**Day 3**	0.20 ±0.01	1.15 ±0.05^b^	1.26 ±0.10^b^	1.23 ±0.05^b^
**Day 5**	0.25 ±0.03	2.00 ±0.08^c^	0.67 ±0.16 ^b,d^	0.48 ±0.05^a,d^
**Day 7**	0.21 ±0.02	2.50 ±0.20^c^	0.30 ±0.12^d^	0 ±0.00^e^

^1^Not-infected only scratched ^2^ Infected by E. coli without treatment ^a^P<0.05, ^b^ P<0.01, ^c^P<0.001 vs. control group; ^d^ P<0.01, ^e^ P<0.001 compared to only infected groups.
